# Optimizing Dose Conversion from IR-Tac to LCP-Tac Formulations in Renal Transplant Recipients: A Population Pharmacokinetic Modeling Study

**DOI:** 10.3390/pharmaceutics17091185

**Published:** 2025-09-12

**Authors:** Zeyar Mohammed Ali, Beatriz Fernández-Alarcón, Pere Fontova, Anna Vidal-Alabró, Raul Rigo-Bonnin, Edoardo Melilli, Nuria Montero, Anna Manonelles, Ana Coloma, Alexandre Favà, Josep M. Grinyó, Josep M. Cruzado, Helena Colom, Nuria Lloberas

**Affiliations:** 1 Nephrology Department, Hospital Universitari de Bellvitge-IDIBELL, 08907 Barcelona, Spain; 2 Biopharmaceutics and Pharmacokinetics Unit, Department of Pharmacy and Pharmaceutical Technology and Physical Chemistry, School of Pharmacy and Food Sciences, University of Barcelona, 08028 Barcelona, Spain; 3 Biochemistry Department, Hospital Universitari de Bellvitge-IDIBELL, 08907 Barcelona, Spain; 4 Medicine Unit, Department of Clinical Sciences, University of Barcelona, 08028 Barcelona, Spain

**Keywords:** tacrolimus, IR-Tac, LCP-Tac, population pharmacokinetics, conversion ratio, CYP3A5, immunosuppression

## Abstract

**Background/Objectives**: Tacrolimus dosing remains challenging due to its narrow therapeutic index and high inter- and intra-patient variability. The extended-release once-daily tacrolimus (LCP-Tac) formulation provides enhanced bioavailability and a sustained pharmacokinetic profile compared to the immediate-release twice-daily tacrolimus (IR-Tac) formulation. Although a general conversion ratio of 1:0.7 is widely recommended when switching between formulations, current guidelines do not account for pharmacogenetic variability. This study aimed to determine whether CYP3A5 genotype influences the conversion ratio in Caucasian renal transplant recipients using population pharmacokinetic (PopPK) modeling. **Methods**: A PopPK model was developed in NONMEM using full PK profiles (10–18 samples per patient) from 30 stable renal transplant patients treated with both IR-Tac and LCP-Tac. **Results**: Tacrolimus pharmacokinetics were best described by a two-compartment model with first-order absorption and linear elimination with distinct absorption rate constants and lag times for each formulation. Including circadian rhythm in the apparent clearance (CL/F) and Ka of IR-Tac significantly improved the model. CYP3A5 polymorphism was the most powerful covariate explaining variability on CL/F. CYP3A5*1 expressers showed higher clearance and lower exposure requiring a more pronounced dose reduction upon conversion to LCP-Tac. Simulations indicated optimal conversion ratios of 1:0.6 for CYP3A5*1 expressers and 1:0.7 for non-expressers. **Conclusions**: These findings highlight the need to move beyond a one-size-fits-all conversion ratio and adopt genotype-informed strategies. LCP-Tac’s enhanced bioavailability requires dose reduction, greater in expressers when switching from IR-Tac. These genotype-specific recommendations provide clinically actionable guidance to complement therapeutic drug monitoring and support more individualized conversion protocols in renal transplantation.

## 1. Introduction

Optimizing tacrolimus (Tac) dosing in transplant patients remains a significant challenge due to the drug’s narrow therapeutic window and high inter- and intra-individual pharmacokinetic (PK) variability [1,2]. This variability translates to difficulties in achieving optimal drug concentrations, which could lead to increased risk of graft rejection or toxicity [2,3,4].

Originally, tacrolimus was formulated as a twice-daily immediate-release formulation (IR-Tac), which offers rapid absorption from the gastrointestinal and a PK profile with high fluctuation between peak (C_max_) and trough (C_trough_) concentrations [2,5,6,7]. LCP-Tac formulation was developed to increase bioavailability and reduce the PK fluctuations. The melt-dose technology enhances the oral bioavailability of the poorly water-soluble tacrolimus allowing for a gradual release along the entire gastrointestinal tract from the small intestine to the colon [5,8,9,10,11,12,13]. The different release profiles between both formulations significantly impact drug absorption requiring a lower dose of LCP-Tac than IR-Tac to achieve similar total tacrolimus exposure in the bloodstream [8,14,15]. Indeed, the current European guidelines recommend a conversion ratio of 1:0.7 for the daily tacrolimus dose between IR-Tac and LCP-Tac, regardless of the genetics [16].

Tacrolimus is primarily metabolized by cytochrome P450 enzymes CYP3A5 and CYP3A4 in the liver and gut [2,17,18,19,20]. However, the intrinsic metabolic capacity of CYP3A5 predominates over that of CYP3A4 [21]. CYP3A5 genetic polymorphism is a key factor influencing the wide variability in tacrolimus exposure observed among patients [2,22,23], affecting both IR-Tac [24,25] and LCP-Tac [26,27]. In addition to the consensus [28], guidelines for using the CYP3A5 genotype to adjust tacrolimus dosing are only available for IR-Tac [23]. Recently, some publications have described the effect of CYP3A5 polymorphisms [26,27] or CYP3A4/A5 SNPs combined cluster on tacrolimus exposure after LCP-Tac [26,27,29]. Furthermore, dose recommendations focusing on CYP3A5 SNPs have been provided for LCP-Tac [26,27]. However, no suggestions have been published regarding the consideration of CYP3A5 genotype in the dose conversion ratio from IR-Tac to LCP-Tac.

To date, the ASERTAA study is the only crossover study that has compared tacrolimus exposure following IR-Tac and LCP-Tac administration in the same patients. This study included stable African American kidney transplant recipients. No significant differences in tacrolimus exposure were observed between CYP3A5 expressors and non-expressors for either IR-Tac or LCP-tac, when non-expressors received doses that were 20% lower than those administered to expressors. A trend to higher exposures for LCP-Tac compared to IR-Tac was observed in both groups due to the higher bioavailability of the extended-release formulation; however, the study did not provide conclusive evidence regarding potential differences in the dose ratio of LCP-Tac to IR-Tac based on genotype. No conversion studies have exclusively focused on a Caucasian population. Due to variation in SNPs prevalence across racial groups, the ASERTAA results might not fully represent the Caucasian population. Additionally, while several population pharmacokinetic (PopPK) models have been developed to study the effects of CYP3A SNPs on IR-Tac [24] and LCP-Tac [26,27], no PopPK study has focused on the conversion.

Our previous study [30] showed that conversion of IR-Tac to LCP-Tac with a unique conversion ratio for CYP3A5 phenotypes does not result in the same tacrolimus exposures. Specifically, CYP3A5*1 expressers exhibited higher tacrolimus exposures with LCP-Tac compared to IR-Tac despite receiving approximately 30% lower doses (dose conversion ratio 1:07). In contrast, non-expressers showed similar exposures between LCP-Tac and IR-Tac with the same conversion ratio. Consequently, results suggest an individualization for the conversion ratio depending on the CYP3A5 metabolizer phenotype.

Given the previous results, a population pharmacokinetic model incorporating data from both formulations in the same patients accounting for key predictors of variability in tacrolimus pharmacokinetics such as genetics, circadian rhythm as well as age, body composition and biochemical variables could be a useful tool to establish the conversion ratio to achieve optimal drug exposures. Our study aimed at developing a population pharmacokinetic model specifically investigating the conversion ratio from IR-Tac to LCP-Tac in stable renal transplant patients, and the factors that might influence it, with a special emphasis on genetic variations.

## 2. Methods

### 2.1. Study Design

This study was carried out at Bellvitge University Hospital (Barcelona) following approval by the Clinical Research Ethics Committee (ref. PR175/18) and in accordance with the Declaration of Helsinki. All participants gave written informed consent. Thirty kidney transplant recipients were enrolled in an open-label, prospective, non-randomized, investigator-initiated, single-center clinical trial (clinicalTrials.gov NCT02961608). Eligible subjects had received their transplant at least six months before inclusion and were maintained on an immunosuppressive regimen of tacrolimus, mycophenolate mofetil and prednisone. Subjects were converted from oral twice-daily IR-Tac (Prograf; Astellas Pharma Europe Ltd., Staines, UK), to once-daily LCP-Tac oral (Envarsus; Chiesi Farmaceutici, Parma, Italy). Exclusion criteria included pregnancy or lactation, active infection, HIV, neoplasms, severe gastrointestinal disease, hepatitis B or C, and concurrent use of medications with known interactions with the CYP3A enzyme.

### 2.2. Blood Sampling and Data Recording

For each subject, between 10 and 18 blood samples were collected over two separate 24 h periods, one prior to and one following conversion. The PK profile for both formulations were obtained at steady state, one week before conversion for IR-Tac and four weeks after conversion for LCP-Tac. Samples were collected following the following time points: pre-dose, and at 0.5, 1, 1.5, 2, 3, 4, 6, 8, 12, 12.5, 13, 13.5, 14, 15, 20, and 24 h after administration of IR-Tac or LCP-Tac.

Tacrolimus daily doses and patient demographic data were extracted from medical records at treatment initiation. Hematocrit (%) and serum creatinine concentrations (µmol·L^−1^) were recorded at each sampling occasion. Clinical outcomes assessed included renal function (eGFR), estimated using the chronic kidney disease epidemiology collaboration formula, delayed graft function (DGF), and graft loss.

### 2.3. Tacrolimus Measurement

Tacrolimus concentrations were determined using a previously developed and validated LC-MS/MS method [31]. Chromatographic separation was performed using the Acquity (^®^) UPLC (^®^) (Waters Corporation, Milford, MA, USA) with a C18 BEH™ reversed phase column (2.1 × 50 mm id, 1.7 μm). The lower limit of quantitation was set at 1.0 ng/mL.

### 2.4. Genotyping

Genomic DNA was isolated from a peripheral whole-blood samples using Maxwell RSC^®^ (Promega Corporation, Sydney, Australia) and stored at −80 °C. Genotyping of the CYP3A5*3 G > A (rs776746) polymorphism (SNPs) was carried out using TaqMan SNP Genotyping Assay with the 7900HT Fast Real-time PCR System, Applied Biosystems (Thermo Fisher Scientific, Waltham, MA, USA).

### 2.5. Statistical Analysis

Demographic and biochemical continuous variables are summarized in Table 1 as means with interquartile ranges. Categorical variables, including clinical and genetic characteristics, are presented as counts and percentages. Trough concentrations (Ctrough) correspond to samples collected immediately prior to each dose were reported as geometric means and interquartile ranges. The area under the curve from 0 to 24 h (AUC24) was calculated using the linear-log trapezoidal rule of the non-compartmental analysis with PKNCA package in R [32]. Dose-normalized AUC24 and Ctrough were also calculated and are presented in Table 2 as geometric means with interquartile ranges. The ratio of dose-normalized population geometric means (IR-Tac/LCP-Tac) for AUC24 was calculated as follows:

(1) $${F=([AUC]}_{\left(24IR-Tac\right)}/Dose)/({\left[AUC\right]}_{\left(24LCP-Tac\right)}/Dose)$$

Data were log-transformed prior to analysis. Differences between geometric means were then calculated, and back-transformation was applied to obtain ratio, as previously described [33].

F values were compared statistically with an unpaired *t*-test considering the genetic variant as fixed factor log-transformed values of F were used according to normal practice [34]. R package (ver4.0.3) was used in all the statistical comparisons and statistical significance was set to *p* < 0.05.

### 2.6. Population Pharmacokinetic Analysis

Population pharmacokinetic (PopPK) analysis was performed with the nonlinear mixed-effects modeling approach using NONMEM^®^ version 7.5 (ICON Development Solutions, Hanover, MD, USA). Perl-Speaks-NONMEM (PsN) version 5.2.6 (Uppsala University, Uppsala, Sweden), R version 4.0.3 (R Core Team, Vienna, Austria), Pirana Modelling Workbench version 3.0 (Certara L.P. (Pharsight), St. Louis, MO, USA), and Xpose 4.7.2 (Uppsala University, Uppsala, Sweden) were used for data management, exploratory data analysis, graph plotting and model evaluation. The first-order conditional estimation (FOCEI) method with interaction was used throughout the modelling process.

#### 2.6.1. Base Model Development

The one- and two-compartment open models with linear elimination were tested. First-order oral absorption with or without lag time, as well as transit compartment models, were tested to describe the absorption processes. Inter-individual variability (IIVs) and inter-occasion variability (IOVs) were tested in all PK parameters assuming a log-normal distribution. The models were parameterized in terms of apparent blood elimination clearance (CL/F), apparent central and peripheral compartment distribution volumes (Vc/F and Vp/F), apparent inter-compartmental clearance (CL_D_/F), absorption rate constant (Ka), and lag time (ALAG) for classical lag time models. For transit compartment models, mean transit time (MTT) and the number of compartments (NN) were used. Due to the different absorption profiles of each formulation (IR-Tac and LCP-Tac), two different absorption rate constants, and lag times were tested. Bioavailability (F) could not be estimated due to the lack of intravenous data. F value was fixed to 1 for the combination of formulation and genetic variant group taken as reference. In the other cases, the relative bioavailability with respect to the reference group was estimated as follows:

(2) F=1·θx

where θx is the fraction representing the actual relative bioavailability value of formulation genetic variant x with respect to the reference.

In addition, as in our previous study [35], the modelling of the effect of 24 h-circadian rhythms on the PK parameters of IR-Tac, previously reported [36], was tested. Thus, as before, a cosine function with a period of 24 h (1440 min) was implemented in the model as follows [37]:

(3) P=θ1 + θAMP·COS(2π/1440)·(TIME − θACROPHASE))  

where P represents the studied PK parameter on which the influence of circadian rhythms is tested (i.e., CL/F and Ka), θ1 the mesor (individual value of the PK parameter around which it oscillates), θAMP the amplitude, and ACROPHASE the acrophase (time of the peak of the cosine function). TIME represents the time in minutes starting at midnight of the first PK profile.

Additive, proportional, and combined error models were tested to characterize residual error (RE) variability. Hierarchical models were compared using the likelihood ratio test, based on reductions in the minimum objective function value (MOFV), with statistical significance defined as *p* < 0.005 (ΔMOFV = −7.879 for 1 degree of freedom). For the non-hierarchical models, the most parsimonious model with the lowest MOFV according to the Akaike information criterion (AIC) was selected [38]. Additional criteria considered in model selection and evaluation included decreases in MOFV, parameter precision expressed as percentage relative standard error (RSE%), reductions in inter-individual variability (IIV), η- and ε-shrinkage values [39], model convergence status, condition number, and visual assessment of goodness-of-fit plots.

#### 2.6.2. Covariate Analysis

All physiologically plausible covariates were evaluated for effects on model parameters by first plotting empirical Bayes estimates of the pharmacokinetic parameters versus each covariate and then testing them in NONMEM.

Covariates were initially screened univariately in the model followed by cumulative forward inclusion/backward elimination procedures. During forward addition a significance level of 5% (reduction in the MOFV of >3.841 points) was used, and during backward elimination a significance level of 1% (MOFV increase >6.635 points) was applied. A covariate was considered clinically relevant for a given PK parameter if its inclusion reduced IIV associated with the parameter by at least 10%. All assessed relationships between PK parameters and continuous covariates were mean-centered, and the relationships with parameters were tested as linear, allometric or power functions.

Demographic and biochemistry variables considered physiologically or clinically relevant, including age, gender, total body weight, body mass index, and hematocrit were tested for influence on tacrolimus pharmacokinetics, and the effect of CYP3A5 phenotype was also examined.

Because the primary objective was accurate individual tacrolimus parameter estimation, Bayesian shrinkage was calculated for each parameter in the final model using [39]:

(4) shrinkage=1−(SDηparameter/Ωparameter)

where SDηparameter is the standard deviation of the individual estimates of η (interpatient variability random effect) for each parameter and Ωparameter is the estimate of the standard deviation of the estimated population variance. High shrinkage indicates generally poor individual parameter estimates.

### 2.7. Model Evaluation and Internal Validation

Goodness-of-fit plots were analyzed throughout the modelling process to assess the descriptive capability of the model. The predictive capability was evaluated using prediction-corrected visual predictive checks (pcVPC) based on 1000 simulations [40]. The median and 5th and 95th percentiles of the simulated data and their respective 95% prediction intervals were calculated and visually compared with the same percentiles obtained from the original raw data. A non-parametric resampling bootstrap procedure with replacement, of 1000 replicates from the original dataset was performed to further evaluate the stability and precision of the model parameters. Also, npde (normalized prediction distribution errors) diagnostics were performed [41]. Model adequacy was also evaluated by checking the evenly distribution of predicted discrepancies and comparing the shape, location and variance of distribution parameters to the theoretical normal distribution.

### 2.8. Simulations

The final estimated fixed- and random-effects parameters were fixed in the model to stochastically simulate 1000 time-concentration profiles for IR-Tac and LCP-Tac in CYP3A5*1 expresser and CYP3A5*1 non-expresser patients. Concentrations at steady-state (after at least 10 days of treatment) from a wide range of IR-Tac (from 0.5 to 10 mg) and LCP-Tac doses were simulated with conversion ratios between both formulations ranging from 0.5 to 1 in steps of 0.1. A 12 h dosing schedule (twice a day) for IR-Tac formulation and 24 h dosing schedule for LCP-Tac were simulated. From these simulations, the AUC_24_ and C_trough_ were calculated using the non-compartmental analysis with PKNCA package in R. Thus, 1000 sets of IR-Tac and LCP-Tac AUC_24_ and C_trough_ values for each IR-Tac dose/CYP3A5 genotype (CYP3A5*1 expressers and non-expressers)/conversion ratio from IR-Tac to LCP-Tac combination were generated. Then, geometric means and 90% confidence intervals were calculated for each set of values of each combination using the R software (ver4.0.3).

## 3. Results

### 3.1. Patient Characteristics and Datasets

A total of 932 blood tacrolimus concentration-time data from 30 stable adult renal transplant recipients were simultaneously analyzed: 481 samples were obtained 1 week before conversion when patients were taking IR-Tac. The remaining 451 samples were obtained 4 weeks after the patients were converted to the LCP-Tac formulation. Demographic, laboratory, and genetic characteristics of the patients are summarized in Table 1. Patients showed mean body weights around 70 kg and mean ages around 60 years, without a wide range of variation between minimum and maximum values.

Renal functions and hematocrit levels were within the expected values for stable kidney transplant patients. Only one patient of CYP3A5 *1/*1 genotype was available in the study and could not be included in the analysis. Instead, we categorized both CYP3A5 *1/*1 and CYP3A5 *1/*3 (N = 10) as expressers and CYP3A5 *3/*3 (N = 20) as non-expressers for the statistical analysis and model development.

Upon conversion from the IR-Tac to LCP-Tac formulation, the normalized by dose exposure increases significantly. The relative bioavailability of IR-Tac vs. LCP-Tac based on AUC_24_ was different between CYP3A5*1 expressers and non-expressers; specifically, the relative bioavailability for CYP3A5*1 expressers was 60%, whereas for non-expressers was of 72% (Table 2).

### 3.2. Population PK Analysis

#### 3.2.1. Base Model

The tacrolimus PK was best described by a two-compartment model with first-order absorption and linear elimination for both formulations. IIV could be associated with CL/F, Vc/F, and with both Ka of each formulation assuming a log-normal distribution. A partial OMEGA block structure with an OMEGA block on Vc/F, Ka IR-Tac, and Ka LCP-Tac was the most appropriate structural model. Inclusion of IOV in CL/F resulted in a statistically significant reduction of the OFV (∆MOFV = −246 units). Similarly, inclusion of IOV in Vc/F reduced the MOFV by 146 units and contributed to a 33% reduction IIV associated with Vc/F.

Two distinct Ka and lag time values were estimated for each formulation, both of which were statistically significant leading to reductions in MOFV of −411 and −196 units, respectively. Considering two ka also reduced inter-individual variability associated with this parameter by 47%. The peripheral compartment distribution volume had to be fixed to the estimated amount from the model, a value which is similar with our previous model [42]. This approach was employed to increase the estimation precision of the remaining parameters of the model and to avoid collinearities. The proportional error model best described the residual error associated with concentrations. Inclusion of IOV in CL/F and Vc/F improved RE by 17% and 13%, respectively. The estimation of lag-time, together with inclusion of circadian rhythm effects on the IR-Tac absorption rate constant, further reduced residual error by 13% and 14%, respectively.

Incorporation of the circadian rhythm variation in the apparent elimination clearance and in the absorption rate constant of the IR-Tac formulation significantly improved the model leading to reductions of the MOFV of −56 units and −195 units, respectively.

#### 3.2.2. Covariate Model

Graphical exploration of Bayesian estimates of the pharmacokinetic parameters vs. demographic and biochemical covariates did not show any significant trend. When covariates were entered univariately, none of the size descriptors (body weight, body mass index) entered allometrically or with any other relationship provided a significant drop in the MOFV (*p* > 0.05) or improved the overall model. Similarly, this, occurred with age and hematocrit.

**Figure 1:**
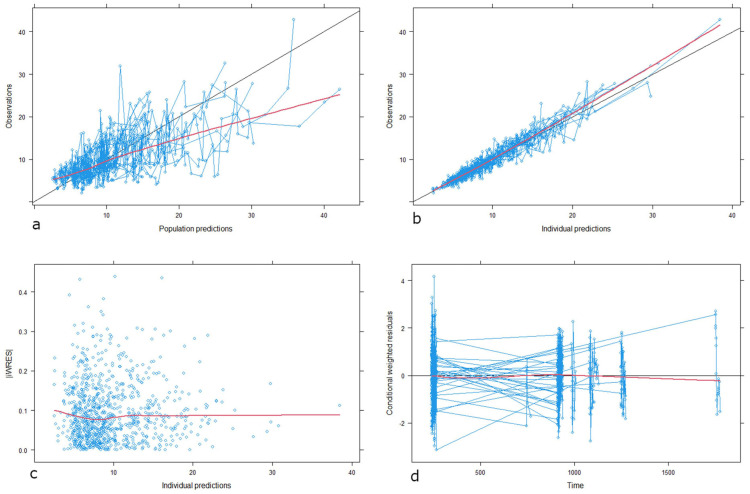
Goodness-of-fit plots of the final model. (**a**) Observed tacrolimus concentrations vs. population predicted concentrations. (**b**) Observed tacrolimus concentrations vs. individual predicted concentrations. Black line: identity line; red line: smooth line indicating the general data trend. (**c**) Individual weighted residuals (IWRES) vs. individual predicted concentrations. (**d**) Conditional weighted residuals (CWRES) vs. time from the start of the study. Red line: smooth line indicating the general data trend. Black line represents the line y  =  0. Time in hours. Concentrations given in ng·mL^−1^.

The influence of CYP3A5 genotypes, categorized as CYP3A5*1 expressers (i.e., *1/*3, and *1/*1)) and non-expressers (CYP3A5*3/*3) was tested in F and CL/F. Statistical significance was superior when tested in F than *CL/F*. Indeed, the inclusion in F significantly improved the model by decreasing the OFV (∆MOFV = −51 units). In addition, it resulted in an overall improvement in the IIV of the most parameters of the model by about 10%. It led to a reduction in unexplained IIV associated with CL/F, Vc/F and Ka by more than 30%, 10% and 8%, respectively. Then, the final model only supported the inclusion of CYP3A5 polymorphisms as a covariate in F. Model performance was further evaluated through goodness-of-fit (GOF) plots, which are presented in Figure 1. GOF plots confirmed the descriptive capability of the data as the observed versus both population and individual predicted concentrations showed a random distribution around the identity line without bias and trends. Conditional weighted residuals were homogeneously spread around zero over all the time after dose range, suggesting that the structural part of the model was well described. Individual weighted residuals were also randomly scattered around zero over the concentration range suggesting a good description of residual error.

The final population pharmacokinetic parameters are displayed in Table 3. The final model estimated the relative bioavailability of CYP3A5*1 expressers-IR-Tac, CYP3A5*1 expressers-LCP-Tac and CYP3A5*1 non-expressers-IR-Tac with respect to CYP3A5*1 non-expressers-LCP-Tac resulting in values of 42.7%, 69.3% and 74.5%, respectively. Within each genetic variant (CYP3A5*1 expressers or non-expresser), bioavailability was significantly higher for LCP-Tac than IR-Tac. Within each formulation, bioavailability was lower for CYP3A5*1 expressers than for non-expressers.

Residual error variability associated with the final model was 13.3%, and the corresponding shrinkage was 9.95%. Most of the parameters were estimated precisely (relative standard error, %RSE < 20%); the absorption rate constant of the IR-Tac formulation and the amplitude of the circadian rhythm variation in it had higher %RSE (~40%). The shrinkage of the IIV related to parameters were within acceptable values (<26%).

#### 3.2.3. Model Evaluation

**Figure 2:**
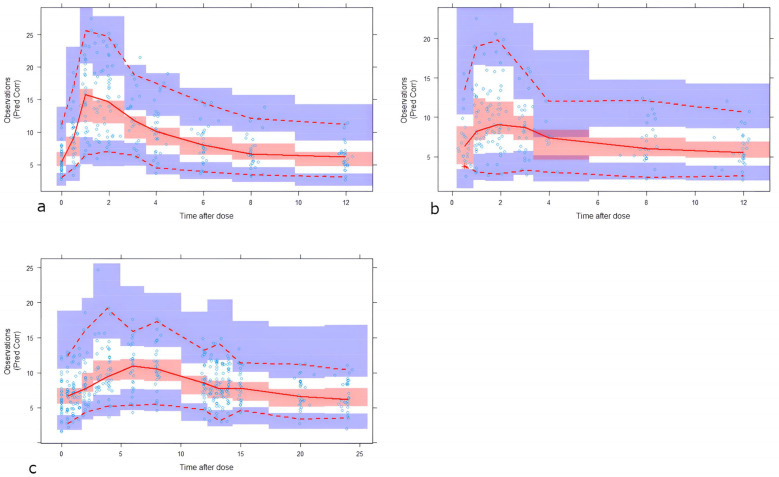
Prediction-corrected visual predictive check (pcVPC) for the final model. (**a**), IR-Tac day dose. (**b**), IR-Tac night dose. (**c**), LCP-Tac. Tacrolimus concentration given in ng·mL^−1^, time after dose given in hours. The solid line represents the median observed prediction-corrected whole blood concentrations (ng·mL^−1^), and the red band represents a simulation-based 95% confidence interval for the median. The observed 5% and 95% percentiles are presented with dashed red lines, and the 95% confidence intervals for the corresponding model predicted percentiles are shown as blue bands. The observed whole blood concentrations (prediction corrected in the pcVPC) are represented by blue circles.

According to the bootstrap results (Table 3) the mean values of all the fixed effect and random effect parameters were within 90% confidence interval of those obtained by the final model confirming the reliability of them. Model parameters were identifiable from the data as indicated by the corresponding 90% confidence interval which did not include the zero. The Predcorr VPC (Figure 2) showed that the model properly describes the mean tendency and variability of the entire data.

The scatter plots of NPDE vs. time and individual predicted concentrations (Figure S1) showed a random distribution around the null line with most of the predicted NPDE values within the 95% confidence interval of the theoretical normal distribution, proving the descriptive capability of the model.

### 3.3. Model Simulations

**Figure 3:**
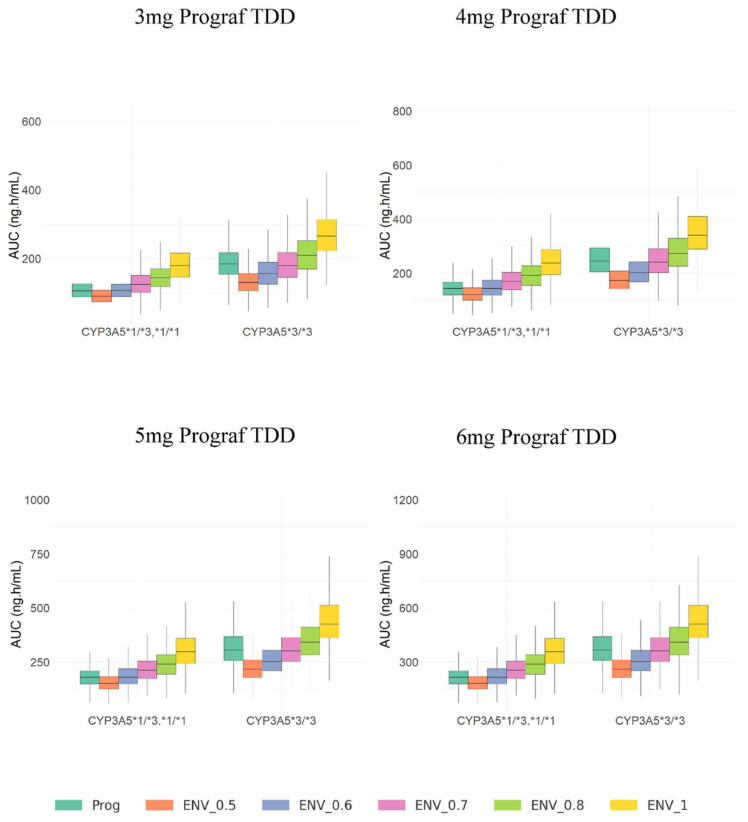
Boxplots of simulated areas under the curve at steady-state conditions (AUC24) for IR-Tac and LCP-Tac after IR-Tac total daily doses from 3 to 6 mg (equivalent to 0.042 to 0.085 mg/kg based on 70 kg of bodyweight, in steps of 1 mg). Colors indicate conversion ratios from 0.5 to 1 (in steps of 0.1). Lower and upper box limits represent the first and the third quartile. Outliers are not shown. The middle solid line is the median.

**Figure 4:**
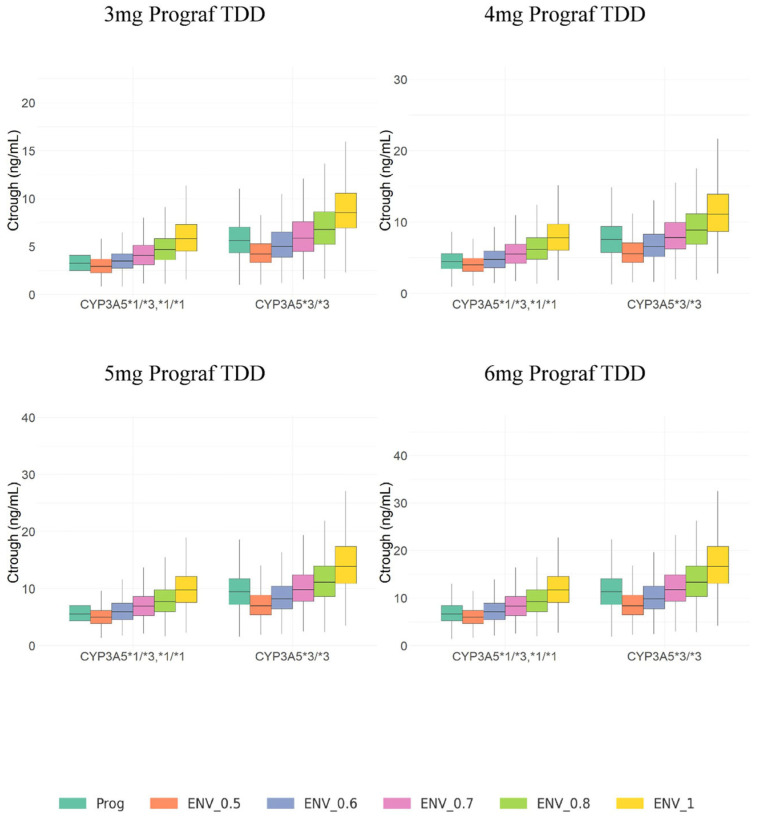
Boxplots of simulated C_trough_ values at steady-state conditions for IR-Tac and LCP-Tac after IR-Tac total daily doses from 3 to 6 mg (equivalent to 0.042 to 0.085 mg/kg based on 70 kg of bodyweight, in steps of 1 mg). Colors indicate conversion ratios from 0.5 to 1 (in steps of 0.1). Lower and upper box limits represent the first and the third quartile. Outliers are not shown. The middle solid line is the median.

Figure 3 and Figure 4 display the boxplots of AUC_24_ and C_trough_ simulated values for IR-Tac and LCP-Tac at steady-state after IR-Tac doses from 3 to 6 mg in steps of 1 mg (corresponding to 0.042 mg/kg to 0.085 mg/kg, for a 70 kg-based bodyweight corresponding to the mean of the studied population) and conversion ratios from IR-Tac to LCP-Tac from 0.5 to 1, in steps of 0.1. These simulations were performed for patients CYP3A5*1 expressers and non-expressers.

Table S1 displays the geometric means (90% confidence intervals) of C_trough_ and AUC_24_ for each IR-Tac dose and conversion ratio from IR-Tac to LCP-Tac and CYP3A5 genetic variant.

According to these results, within the same dose lower tacrolimus exposures given by either C_trough_ or AUC_24_, are achieved for IR-Tac in CYP3A5*1 expressers compared to non-expressers. In addition, regardless of the conversion ratio, tacrolimus exposures for LCP-Tac are also lower in CYP3A5*1 expressers compared to non-expressers. On the other hand, tacrolimus exposures for LCP-Tac increase with the conversion ratio applied. This behavior is observed within each dose regimen but, as expected, tacrolimus exposures for both IR-Tac and LCP-Tac increase proportionally with dose.

Comparison of simulated C_trough_ and AUC_24_ values for IR-Tac (0–24 h) and LCP-Tac, CYP3A5 expressers have higher tacrolimus exposure with LCP-Tac compared to IR-Tac when the LCP-Tac dose is reduced by 30%, whereas non-expressers show similar exposures between LCP-Tac and IR-Tac with the same conversion ratio. This suggests the optimal conversion ratio at steady state for CYP3A5*1 non-expresser is 0.70; meanwhile, a conversion ratio of 0.60 should be applied for expressers to achieve similar exposures.

## 4. Discussion

This study marks a significant advancement in the understanding of tacrolimus pharmacokinetics in stable renal transplant patients, particularly in the context of conversion from IR-Tac to LCP-Tac. Notably, the importance of our research lies in the introduction of the first PopPK model that specifically investigates the dose conversion ratio of tacrolimus between these formulations based on genetic polymorphism in a stable renal transplant population.

The model developed confirms the findings of our previous study [30]. Certainly, the model showed that the standard conversion ratio from IR-Tac to LCP-Tac, at steady-state conditions, proposed by current guidelines [16] (0.7–0.8 of the IR-Tac dose) to achieve similar tacrolimus daily exposures between both formulations would not be appropriate for both genotypes of CYP3A5 (CYP3A5*1 expressers vs. non-expressers). This is particularly important considering that there are few conversion studies [5,10] which were either conducted solely in African American patients or in a mixed population of Caucasian and non-Caucasian individuals.

As previously [24,27,42,43,44], the tacrolimus pharmacokinetic profile was best described by a two-compartment model with delayed first order absorption. Previous studies [27,42], used transit compartment models to better describe delayed absorption. However, in our case they were not supported by the data, probably due to overparameterization. This would explain the differences in the absorption rate constant Ka for IR-Tac and for LCP-Tac formulations when comparing the current model (Ka: 2.04 and 0.111 h^−1^ for IR-Tac and LCP-Tac, respectively) to previous (Ka: 0.47 and 0.72 h^−1^ for IR-Tac [27,42] and LCP-Tac [27,42], respectively). Similar results occurred with lag-times that were 0.465 and 1.42 h for IR-Tac and LCP-Tac, respectively, in our model and 2.49 and 5.82 h for IR-Tac [27,42] and LCP-Tac [27,42] formulations in earlier studies.

In any case, the intensive sampling scheme allowed an adequate description of the whole PK profile for both day and night IR-Tac administrations and during the 24 h LCP-Tac period. Unlike previously [24,27], the inclusion of data from only 30 patients who were also stable did not allow the influence of covariates other than the CYP3A5 genetic polymorphism on apparent bioavailability of each formulation (IR-Tac and LCP-Tac) to be identified. This led to different relative apparent bioavailability of IR-Tac versus LCP-Tac between CYP3A5*1 expressers (61.6%) and non-expressers (74.5%); these values are in agreement with those obtained through the non-compartmental analysis (Table 2). According to the estimated relative F values (Table 3), the model provided apparent elimination clearance values of 11.9, 15.97, 17.17 and 27.87 L/h for CYP3A5*1 non-expresser-LCP-Tac, CYP3A5*1 non-expressers-IR-Tac, CYP3A5*1 expressers-LCP-Tac, and CYP3A5*1 expressers-IR-Tac, respectively, these values being in line with previous studies [27,45]. All these results supported the descriptive capability of the model; meanwhile, the visual predictive checks confirmed its predictive capability. Simulations from the final model (Table S1) confirmed that regardless of the administered dose, a conversion ratio of 1:0.7 is required for CYP3A5*1 non-expressers to achieve similar exposures between both formulations, while the conversion ratio 1:0.6 is enough for CYP3A5*1 expressers. For patients with higher clearance such as CYP3A5*1 expressers, the initial dose needed to achieve target steady-state concentrations with the IR-Tac formulation was higher. With the same IR-Tac dose regimen, lower exposures were achieved in CYP3A5*1 expressers compared to non-expressers (Figure 3 and Figure 4). When switching to LCP-Tac with increased bioavailability (F), the fractions of Tac that enter circulation increase and because steady-state concentrations are proportional to F, higher F results occur for the same dose regimen in higher exposure, this explaining the required dose reduction in all the patients. However, patients with higher clearance such as CYP3A5*1 expressers require a lower LCP-Tac dose than non-expressers to maintain the lower steady-state concentrations achieved after the IR-Tac compared to non-expressers. This is a key focus of the current study.

This finding should also be taken into account for other patients with high clearance, such as those receiving concomitant treatment with corticosteroids, also requiring careful dose adjustments to maintain the previous steady-state drug levels.

Consequently, once the steady-state for a given dosage regimen of IR-Tac is achieved, dose requirements of LCP-Tac are different between both genotypes, i.e., CYP3A5*1 expressers require a 40% lower dose of LCP-Tac than IR-Tac; meanwhile, for non-expressers, the LCP-Tac dose should be 30% less than that of IR-Tac (Table S1). In contrast, ASERTAA found no AUC_0–24_ difference between CYP3A5*1 expressers and non-expressers for either formulation, which likely reflects key differences in the ASERTAA study such as enrollment of predominantly African American recipients (76% expressers), shorter post-transplant intervals, higher tacrolimus doses, and inclusion of additional nonfunctional CYP3A5 variants [10]. As expected, regardless of the conversion ratio used, tacrolimus exposure at steady state with the modified-release formulation (LCP-Tac) remains lower in CYP3A5*1 expressers than in non-expressers, regardless of the administered dose of IR-Tac.

The observed higher AUC_0–24_ for LCP-Tac compared to IR-Tac, and the lower AUC_0–24_ for CYP3A5*1 expressers compared to non-expressers within the same formulation, align with findings from previous studies [5,10,13,27,46]. Several factors can contribute to the higher bioavailability of LCP-Tac compared to IR-Tac but the most important is the used MeltDose™ drug-delivery technology, which enhances oral bioavailability, controls drug release, and produces a more distal distribution of tacrolimus within the gut [9,10]. In the ASERTAA study, the authors suggested that the minor susceptibility of LCP-Tac to the CYP3A5 genotype may be because LCP-Tac is absorbed in the more distal gastrointestinal tract where CYP3A5 activity is decreased [47,48,49]. However, regional distribution of CYP3A5 seems not to have a relevant impact on bioavailability [49].

The limitation of our study lies in the relatively small sample size, underscoring the need for larger clinical trials to validate and strengthen our findings. Additionally, our model could not explore the cluster genotype combination of CYP3A4 and CYP3A5 due to data limitations. It is important to note that this cluster has previously been demonstrated to play a crucial role in contributing to the clearance variation of tacrolimus in both formulations, extending beyond the influence of CYP3A5 alone [24,27]. Future research endeavors should aim to incorporate this aspect for a more comprehensive understanding of the pharmacogenetic factors influencing tacrolimus pharmacokinetics.

Our results provide clinically relevant insights for practice. While current guidelines propose a fixed conversion ratio irrespective of genotype, our study is the first to provide quantitative PopPK evidence in a Caucasian cohort that genotype should inform conversion. This directly challenges the current ‘one-size-fits-all’ approach. Incorporating genotype into conversion protocols offers clinicians a more practical starting point for dose adjustment, which, in combination with therapeutic drug monitoring, may help minimize the risks of underexposure and toxicity during formulation switches.

## Figures and Tables

**Table 1:** Demographic, biochemical and clinical characteristics of the patients included in the study.

Characteristics	IR-Tac	LCP-Tac
Patients (*n*)	30	30
Samplings (*n*)	481	451
Gender Male/Female, (*n*/*n*)	22/8	22/8
Weight (Kg)	72 (64–80)	73 (64–80)
Age (Years)	58 (48–68)	58 (48–68)
BMI (Kg·m^−2^)	26 (21.5–29.3)	27 (21.5–29.3)
HTC (%)	40.9 (37.6–44.8)	40.1 (37.1–43)
GFR (mL·min^−1^)	49.6 (34–57)	49.3 (42–58)
Cr (μmol·L^−1^)	141.9 (108–166)	147.6 (111–155)
CYP3A5 Genotype		
*1/*3 n (%)	9 (30%)	9 (30%)
*1/*1 n (%)	1 (3%)	1 (3%)
*3/*3 n (%)	20 (67%)	20 (67%)

Values are given as arithmetic mean (interquartile range) for continuous variables, and as count (*n*) or count and percentage for categorical variables. BMI, body mass index; HTC, hematocrit; GFR, glomerular filtration rate estimated by the CKD-EPI formula; Cr, serum creatinine.

**Table 2:** Comparative C_trough_ and AUC_24_ values sorted by formulation and CYP3A5 genotypes normalized by dose values are also presented.

Formulation/ Genotype Group	Dose (mg·day^−1^)	N	C*_trough_* (ng·mL^−1^)	C*_trough_*/D	AUC_24_ (ng·h·mL^−1^)	AUC_24_/D	Relative Bioavailability	*p*-Value
IR-Tac								
CYP3A5 *1/*1, *1/*3	5 (3–12)	20	4.9 (4.6–5.2)	1.6(1.4–2)	195 (184–224)	32 (27–43)		
LCP-Tac							0.60	<0.001 *
CYP3A5 *1/*1, *1/*3	3.75 (2–8.5)	10	5.6 (4.5–6.7)	1.28 (0.9–1.8)	232 (173–286)	53 (38–71)		
IR-Tac								
CYP3A5 *3/*3	3 (1.5–8)	20	5.7 (4.7–7.2)	3.6 (2.9–4.6)	212 (169–250)	68 (56–81)		<0.001 #
LCP-Tac							0.72	
CYP3A5 *3/*3	2 (1–4.75)	10	5.7 (4.7–6.7)	2.7 (2.2–3.3)	199 (163–265)	94 (76–122)		

AUC24; Area under the blood concentration time-curve from 0 to 24 h. C_trough_: trough blood concentrations. Values are given as geometric means (interquartile range) for AUC_24_ and C_trough_. Doses are expressed as median (range). *p*-values are statistical comparisons for mean AUC/D values, *: differences between IR-Tac and LCP-Tac for CYP3A5 expressers, #: differences between IR-Tac and LCP-Tac for CYP3A5 non-expressers.

**Table 3:** Tacrolimus population pharmacokinetic parameter estimates and bootstrap results for the final model.

		Final Model Parameter Estimates (RSE%)	Bootstrap Results *	
Parameter	Description	Value	Bootstrap Median	90% CI
Disposition PK parameters				
CL/F (L·h**^−1^**)	Apparent Elimination Clearance	11.9 (8.5%)	11.85	10.34–13.53
Vc/F (L)	Apparent Distribution Volume of central compartment	78 (14.7%)	81	63–100.22
CLd/F (L·h**^−1^**)	Apparent Distributional Clearance	25.8 (8.5%)	25.75	22.08–29.39
Vp/F (L)	Apparent Distribution Volume of peripheral compartment	500 FIX	-	-
Absorption parameters				
Ka IR-Tac	Absorption rate constant (IR-Tac)	2.04 (40%)	2.17	1.23–3.72
Ka LCP_Tac	Absorption rate constant (LCP-Tac)	0.111 (16.9%)	0.115	0.08–0.15
F LCP-Tac_PM	Reference group for Relative bioavailability (LCP-Tac_CYP3A5*1 non-expresser)	1 FIX	-	-
F IR-Tac_PM	Relative bioavailability of IR-Tac_CYP3A5*1 non-expresser compared to reference	0.745 (7.6%)	0.757	0.66–0.84
F LCP-Tac_HM	Relative bioavailability of LCP-Tac_CYP3A5*1 expresser compared to reference	0.693 (13.7%)	0.695	0.52–0.85
F IR-Tac_HM	Relative bioavailability of IR-Tac_CYP3A5*1 expresser compared to reference	0.427 (13.4%)	0.428	0.34–0.52
Lag-Time IR-Tac (h)	lag time for IR-Tac formulation in hours	0.465 (0.1%)	0.465	0.42–0.47
Lag-Time LCP-Tac (h)	lag time for LCP-Tac formulation in hours	1.4 (2.4%)	1.39	1.32–1.57
Circadian rhythms parameters				
Acrophase_CL/F_ (h)	peak time of the cosine function	17 (3.6%)	16.94	15.94–17.98
Amp_CL/F_	Amplitude	3.42 (17.1%)	3.41	2.33–4.39
Acrophase_ka_ (h)	peak time of the cosine function	3.13 (18.3%)	3.17	1.82–4.52
Amp_ka_	Amplitude	1.55 (44.5%)	1.64	0.91–2.97
RE. (-)	Combined residual error	13.30 (8.2%)	13.11	11.83–14.14
**Interindividual patient variability**	**Description**	**CV% (RSE%)**		
IIV_CL/F_	IIV associated with Elimination Clearance	26.49 (29.1%)	25.49	18.7–31.14
IIV_Vc/F_	IIV associated with Distribution Volume of central compartment	53.47 (42%)	52.15	33.46–72.20
Vc/F/Ka IR-Tac Correlation	Correlation between IIV of Vc/F and Ka of IR-Tac	75.63 (16%)	72.3	43–92.33
Vc/F/Ka LCP-Tac Correlation	Correlation between IIV of Vc/F and Ka of LCP-Tac	44.38 (10%)	44.11	12.76–65.68
IIV_Ka IR-Tac_	IIV associated with Absorption rate constant (IR-Tac)	150.66 (25.6%)	146.62	87.6–184.44
Ka IR-Tac/Ka LCP-Tac Correlation	Correlation between IIV of Ka IR-Tac and Ka LCP-Tac	45 (20.3%)	41.24	38.69–75.55
IIV_Ka LCP_Tac_	IIV associated with Absorption rate constant (LCP-Tac)	67.23 (46.5%)	72.25	46.96–88.67
IOV_CL_	IOV associated with Elimination Clearance	20.85 (23.9%)	20	16.9–24.51
IOV_Vc_	IOV associated with Distribution Volume of central compartment	58.82 (28.9%)	58.05	38.47–72

RSE: Relative Standard Error; IIV: Inter-Individual Variability; IOV: Inter occasion variability; CV: Coefficient of variation; CI: Confidence interval. * Non-parametric Bootstrap results based on 756 successful resampling from a total of 1000.

## Data Availability

The data presented in this study are available on request from the corresponding author due to privacy or ethical restrictions.

## Supplementary Materials

The following supporting information can be downloaded at: https://www.mdpi.com/article/10.3390/pharmaceutics17091185/s1, Figure S1: NPDE; Table S1: Simulations.

## References

[1] Kuypers D.R.J. (2020). Intrapatient Variability of Tacrolimus Exposure in Solid Organ Transplantation: A Novel Marker for Clinical Outcome. Clin. Pharmacol. Ther..

[2] Staatz C.E., Tett S.E. (2004). Clinical pharmacokinetics and pharmacodynamics of tacrolimus in solid organ transplantation. Clin. Pharmacokinet..

[3] Venkataramanan R., Swaminathan A., Prasad T., Jain A., Zuckerman S., Warty V., McMichael J., Lever J., Burckart G., Starzl T. (1995). Clinical pharmacokinetics of tacrolimus. Clin. Pharmacokinet..

[4] Katari S.R., Magnone M., Shapiro R., Jordan M., Scantlebury V., Vivas C., Gritsch A., McCauley J., Starzl T., Demetris A.J. (1997). Clinical features of acute reversible tacrolimus (FK 506) nephrotoxicity in kidney transplant recipients. Clin. Transplant..

[5] Tremblay S., Nigro V., Weinberg J., Woodle E.S., Alloway R.R. (2017). A Steady-State Head-to-Head Pharmacokinetic Comparison of All FK-506 (Tacrolimus) Formulations (ASTCOFF): An Open-Label, Prospective, Randomized, Two-Arm, Three-Period Crossover Study. Am. J. Transplant..

[6] Wallemacq P.E., Verbeeck R.K. (2001). Comparative clinical pharmacokinetics of tacrolimus in paediatric and adult patients. Clin. Pharmacokinet..

[7] Wallemacq P.E., Furlan V., Möller A., Schäfer A., Stadler P., Firdaous I., Taburet A.-M., Reding R., Clement De Clety S., De Ville De Goyet J. (1998). Pharmacokinetics of tacrolimus (FK506) in paediatric liver transplant recipients. Eur. J. Drug Metab. Pharmacokinet..

[8] Nigro V., Glicklich A., Weinberg J. (2013). Improved Bioavailability of MELTDOSE Once-Daily Formulation of Tacrolimus (LCP-Tacro) with Controlled Agglomeration Allows for Consistent Absorption over 24 Hrs: A Scintigraphic and Pharmacokinetic Evaluation [abstract]. Am. J. Transpl..

[9] Rostaing L., Bunnapradist S., Grinyó J.M., Ciechanowski K., Denny J.E., Silva H.T., Budde K., Envarsus Study Group (2016). Novel Once-Daily Extended-Release Tacrolimus Versus Twice-Daily Tacrolimus in De Novo Kidney Transplant Recipients: Two-Year Results of Phase 3, Double-Blind, Randomized Trial. Am. J. Kidney Dis..

[10] Trofe-Clark J., Brennan D.C., West-Thielke P., Milone M.C., Lim M.A., Neubauer R., Nigro V., Bloom R.D. (2018). Results of ASERTAA, a Randomized Prospective Crossover Pharmacogenetic Study of Immediate-Release Versus Extended-Release Tacrolimus in African American Kidney Transplant Recipients. Am. J. Kidney Dis..

[11] Langone A., Steinberg S.M., Gedaly R., Chan L.K., Shah T., Sethi K.D., Nigro V., Morgan J.C., STRATO Investigators (2015). Switching STudy of Kidney TRansplant PAtients with Tremor to LCP-TacrO (STRATO): An open-label, multicenter, prospective phase 3b study. Clin. Transplant..

[12] Gaber A.O., Alloway R.R., Bodziak K., Kaplan B., Bunnapradist S. (2013). Conversion from twice-daily tacrolimus capsules to once-daily extended-release tacrolimus (LCPT): A phase 2 trial of stable renal transplant recipients. Transplantation.

[13] Fontova P., Colom H., Rigo-Bonnin R., Bestard O., Vidal-Alabró A., van Merendonk L.N., Cerezo G., Polo C., Montero N., Melilli E. (2021). Sustained Inhibition of Calcineurin Activity With a Melt-Dose Once-daily Tacrolimus Formulation in Renal Transplant Recipients. Clin. Pharmacol. Ther..

[14] Bunnapradist S., Ciechanowski K., West-Thielke P., Mulgaonkar S., Rostaing L., Vasudev B., Budde K. (2013). Conversion From Twice-Daily Tacrolimus to Once-Daily Extended Release Tacrolimus (LCPT): The Phase III Randomized MELT Trial. Am. J. Transplant..

[15] Budde K., Bunnapradist S., Grinyó J.M., Ciechanowski K., Denny J.E., Silva H.T., Rostaing L., Envarsus Study Group (2014). Novel Once-Daily Extended-Release Tacrolimus (LCPT) Versus Twice-Daily Tacrolimus in De Novo Kidney Transplants: One-Year Results of Phase III, Double-Blind, Randomized Trial. Am. J. Transplant..

[16] Staatz C.E., Tett S.E. (2015). Clinical Pharmacokinetics of Once-Daily Tacrolimus in Solid-Organ Transplant Patients. Clin. Pharmacokinet..

[17] Lampen A., Christians U., Guengerich F.P., Watkins P.B., Kolars J.C., Bader A., Gonschior A.K., Dralle H., Hackbarth I., Sewing K.F. (1995). Metabolism of the immunosuppressant tacrolimus in the small intestine: Cytochrome P450, drug interactions, and interindividual variability. Drug Metab. Dispos..

[18] Iwasaki K. (2007). Metabolism of tacrolimus (FK506) and recent topics in clinical pharmacokinetics. Drug Metab. Pharmacokinet..

[19] Iwasaki K., Shiraga T., Matsuda H., Teramura Y., Kawamura A., Hata T., Ninomiya S., Esumi Y. (1998). Absorption, Distribution, Metabolism and Excretion of Tacrolimus (FK506) in the Rat. Drug Metab. Pharmacokinet..

[20] Lloberas N., Vidal-Alabró A., Colom H. (2025). Customizing Tacrolimus Dosing in Kidney Transplantation: Focus on Pharmacogenetics. Ther. Drug Monit..

[21] Dai Y., Hebert M.F., Isoherranen N., Davis C.L., Marsh C., Shen D.D., Thummel K.E. (2006). Effect of CYP3A5 polymorphism on tacrolimus metabolic clearance in vitro. Drug Metab. Dispos..

[22] De Jonge H., De Loor H., Verbeke K., Vanrenterghem Y., Kuypers D.R. (2012). In vivo CYP3A4 activity, CYP3A5 genotype, and hematocrit predict tacrolimus dose requirements and clearance in renal transplant patients. Clin. Pharmacol. Ther..

[23] Birdwell K.A., Decker B., Barbarino J.M., Peterson J.F., Stein C.M., Sadee W., Wang D., Vinks A.A., He Y., Swen J.J. (2015). Clinical Pharmacogenetics Implementation Consortium (CPIC) Guidelines for CYP3A5 Genotype and Tacrolimus Dosing. Clin. Pharmacol. Ther..

[24] Andreu F., Colom H., Elens L., van Gelder T., van Schaik R.H.N., Hesselink D.A., Bestard O., Torras J., Cruzado J.M., Grinyó J.M. (2017). A New CYP3A5*3 and CYP3A4*22 Cluster Influencing Tacrolimus Target Concentrations: A Population Approach. Clin. Pharmacokinet..

[25] Woillard J.B., De Winter B.C.M., Kamar N., Marquet P., Rostaing L., Rousseau A. (2011). Population pharmacokinetic model and Bayesian estimator for two tacrolimus formulations--twice daily Prograf and once daily Advagraf. Br. J. Clin. Pharmacol..

[26] Henin E., Govoni M., Cella M., Laveille C., Piotti G. (2021). Therapeutic Drug Monitoring Strategies for Envarsus in De Novo Kidney Transplant Patients Using Population Modelling and Simulations. Adv. Ther..

[27] Mohammed Ali Z., Meertens M., Fernández B., Fontova P., Vidal-Alabró A., Rigo-Bonnin R., Melilli E., Cruzado J.M., Grinyó J.M., Colom H. (2023). CYP3A5*3 and CYP3A4*22 Cluster Polymorphism Effects on LCP-Tac Tacrolimus Exposure: Population Pharmacokinetic Approach. Pharmaceutics.

[28] Brunet M., van Gelder T., Åsberg A., Haufroid V., Hesselink D.A., Langman L., Lemaitre F., Marquet P., Seger C., Shipkova M. (2019). Therapeutic Drug Monitoring of Tacrolimus-Personalized Therapy: Second Consensus Report. Ther. Drug Monit..

[29] Crespo E., Vidal-Alabró A., Jouve T., Fontova P., Stein M., Mocka S., Meneghini M., Sefrin A., Hruba P., Gomà M. (2022). Tacrolimus CYP3A Single-Nucleotide Polymorphisms and Preformed T- and B-Cell Alloimmune Memory Improve Current Pretransplant Rejection-Risk Stratification in Kidney Transplantation. Front. Immunol..

[30] Vidal-Alabró A., Fontova P., Rigo-Bonnin R., Mohammed Ali Z., Melilli E., Montero N., Manonelles A., Coloma A., Grinyó J.M., Cruzado J.M. (2025).

[31] Rigo-Bonnin R., Arbiol-Roca A., de Aledo-Castillo J.M.G., Alía P. (2015). Simultaneous Measurement of Cyclosporine A, Everolimus, Sirolimus and Tacrolimus Concentrations in Human Blood by UPLC–MS/MS. Chromatographia.

[32] Denney W., Duvvuri S., Buckeridge C. (2015). Simple, Automatic Noncompartmental Analysis: The PKNCA R Package. J. Pharmacokinet. Pharmacodyn..

[33] European Medicines Agency (2010). Guideline on the Investigation of Bioequivalence, Rev. 1. https://www.ema.europa.eu/en/documents/scientific-guideline/guideline-investigation-bioequivalence-rev1_en.pdf.

[34] Davit B.M., Chen M.-L., Conner D.P., Haidar S.H., Kim S., Lee C.H., Lionberger R.A., Makhlouf F.T., Nwakama P.E., Patel D.T. (2012). Implementation of a reference-scaled average bioequivalence approach for highly variable generic drug products by the US Food and Drug Administration. AAPS J..

[35] Fernández-Alarcón B., Mohammed Ali Z., Fontova P., Vidal-Alabró A., Rigo-Bonnin R., Melilli E., Montero N., Manonelles A., Favà A., Coloma A. (2025).

[36] Fontova P., Colom H., Rigo-Bonnin R., van Merendonk L.N., Vidal-Alabró A., Montero N., Melilli E., Meneghini M., Manonelles A., Cruzado J.M. (2021). Influence of the Circadian Timing System on Tacrolimus Pharmacokinetics and Pharmacodynamics After Kidney Transplantation. Front. Pharmacol..

[37] Van Rongen A., Kervezee L., Brill M.J.E., van Meir H., den Hartigh J., Guchelaar H.-J., Meijer J.H., Burggraaf J., van Oosterhout F. (2015). Population Pharmacokinetic Model Characterizing 24-Hour Variation in the Pharmacokinetics of Oral and Intravenous Midazolam in Healthy Volunteers. CPT Pharmacomet. Syst. Pharmacol..

[38] Yamaoka K., Nakagawa T., Uno T. (1978). Application of Akaike’s information criterion (AIC) in the evaluation of linear pharmacokinetic equations. J. Pharmacokinet. Biopharm..

[39] Savic R.M., Karlsson M.O. (2009). Importance of Shrinkage in Empirical Bayes Estimates for Diagnostics: Problems and Solutions. AAPS J..

[40] Bergstrand M., Hooker A.C., Wallin J.E., Karlsson M.O. (2011). Prediction-corrected visual predictive checks for diagnosing nonlinear mixed-effects models. AAPS J..

[41] Comets E., Brendel K., Mentré F. (2008). Computing Normalised Prediction Distribution Errors to Evaluate Nonlinear Mixed-Effect Models: The npde Add-On Package for R. Comput. Methods Programs Biomed..

[42] Andreu F., Colom H., Grinyó J.M., Torras J., Cruzado J.M., Lloberas N. (2015). Development of a population PK model of tacrolimus for adaptive dosage control in stable kidney transplant patients. Ther. Drug Monit..

[43] Fernández-Alarcón B., Nolberger O., Vidal-Alabró A., Rigo-Bonnin R., Grinyó J.M., Melilli E., Montero N., Manonelles A., Coloma A., Favà A. (2024). Guiding the starting dose of the once-daily formulation of tacrolimus in “de novo” adult renal transplant patients: A population approach. Front. Pharmacol..

[44] Andrews L.M., Hesselink D.A., van Schaik R.H.N., van Gelder T., de Fijter J.W., Lloberas N., Elens L., Moes D.J.A.R., de Winter B.C.M. (2019). A population pharmacokinetic model to predict the individual starting dose of tacrolimus in adult renal transplant recipients. Br. J. Clin. Pharmacol..

[45] Åsberg A., Midtvedt K., van Guilder M., Størset E., Bremer S., Bergan S., Jelliffe R., Hartmann A., Neely M.N. (2013). Inclusion of CYP3A5 genotyping in a nonparametric population model improves dosing of tacrolimus early after transplantation. Transplant. Int..

[46] Sanghavi K., Brundage R.C., Miller M.B., Schladt D.P., Israni A.K., Guan W., Oetting W.S., Mannon R.B., Remmel R.P., Matas A.J. (2017). Genotype-guided tacrolimus dosing in African-American kidney transplant recipients. Pharmacogenomics J..

[47] Baccarani U., Velkoski J., Pravisani R., Adani G.L., Lorenzin D., Cherchi V., Falzone B., Baraldo M., Risaliti A. (2019). MeltDose Technology vs. Once-Daily Prolonged Release Tacrolimus in De Novo Liver Transplant Recipients. Transplant. Proc..

[48] Baraldo M. (2016). Meltdose Tacrolimus Pharmacokinetics. Transplant. Proc..

[49] Tsunashima D., Kawamura A., Murakami M., Sawamoto T., Undre N., Brown M., Groenewoud A., Keirns J.J., Holman J., Connor A. (2014). Assessment of tacrolimus absorption from the human intestinal tract: Open-label, randomized, 4-way crossover study. Clin. Ther..

