# Supplementary figures and images for: Optimizing Dose Conversion from IR-Tac to LCP-Tac Formulations in Renal Transplant Recipients: A Population Pharmacokinetic Modeling Study
Source: Pharmaceutics. 2025 Sep 12;17(9):1185. doi: 10.3390/pharmaceutics17091185 (PMC12473248; doi:10.3390/pharmaceutics17091185)

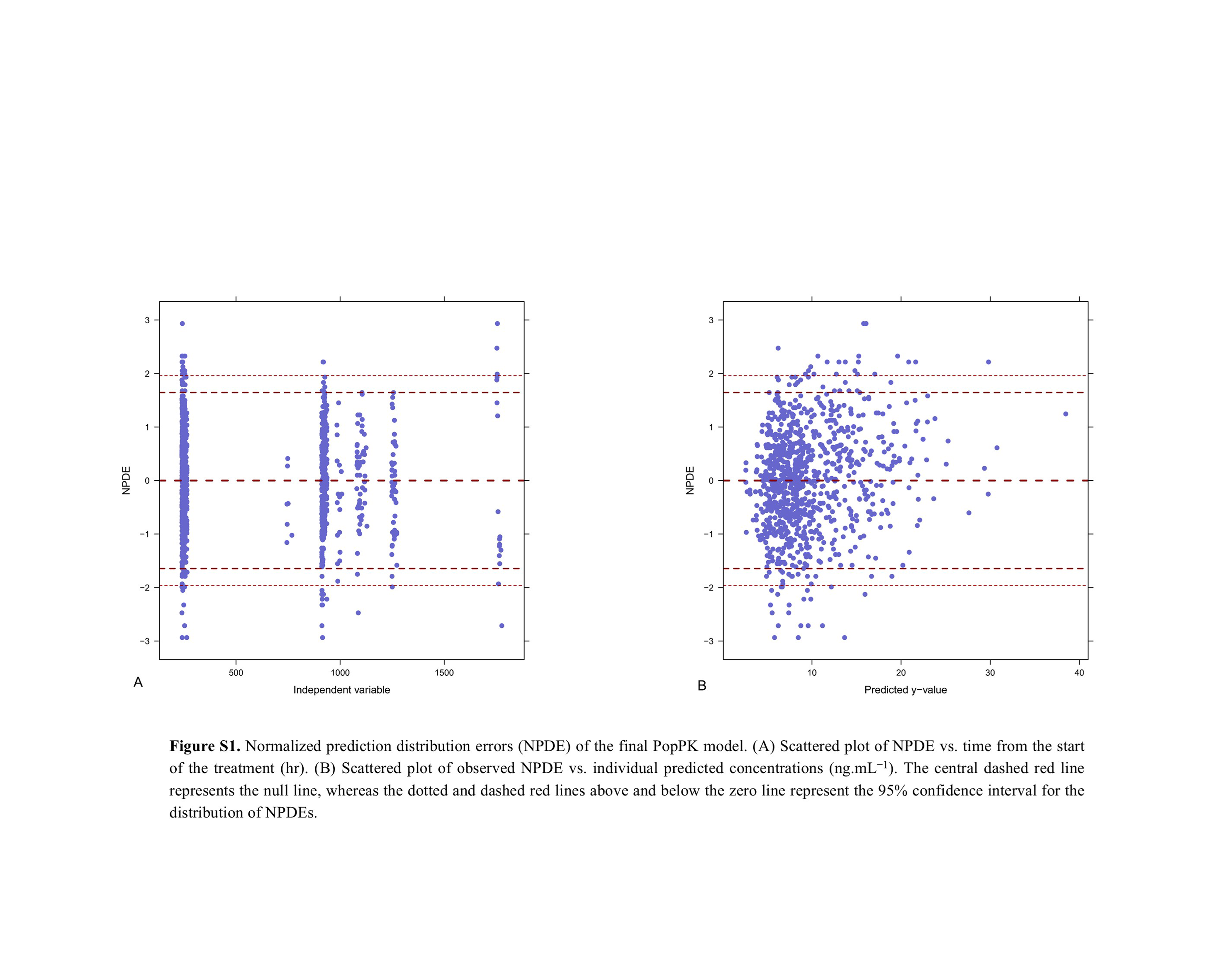

Supplement: Supplementary file 1 [file pharmaceutics-17-01185-s001.zip › Figure S1.tif]
